# The role of red cell distribution width in the diagnosis of acute appendicitis: a retrospective case-controlled study

**DOI:** 10.1186/1749-7922-8-46

**Published:** 2013-11-11

**Authors:** Huseyin Narci, Emin Turk, Erdal Karagulle, Turhan Togan, Keziban Karabulut

**Affiliations:** 1 Department of Emergency Medicine, Baskent Universitesi Konya Hastanesi Hocacihan mah, Saray caddesi No: 1, Selcuklu, Konya 42080, Turkey; 2 Department of General Surgery, Baskent University Faculty of Medicine, Ankara, Turkey; 3 Department of Infectious Diseases and Clinical Microbiology, Baskent University Faculty of Medicine, Ankara, Turkey

**Keywords:** Acute appendicitis, C-reactive protein, Leukocyte, Red cell distribution width

## Abstract

**Purpose:**

The aim of this study was to seek whether red cell distribution width (RDW) has a role in the diagnosis of acute appendicitis. It was also aimed to show the relationship of RDW with leukocyte count and C-reactive protein (CRP) level.

**Methods:**

This study was conducted via retrospective assessment of the hospital records of the adult patients who were operated for acute appendicitis between January 2010 and February 2013 and had a pathology report that confirmed the diagnosis of acute appendicitis. The patients in the control group were selected from healthy adults of similar age who applied to check-up clinic. Age, gender, leukocyte count, CRP, and RDW values were recorded. This study is a case controlled retrospective clinical study.

**Results:**

A total of 590 patients in the acute appendicitis group and 121 patients in the control group were included, making up a total of 711 subjects. The mean RDW levels were 15.4 ± 1.5% in the acute appendicitis group, while 15.9 ± 1.4% in the control group. CRP, leukocyte count were significantly higher in the acute appendicitis group, and RDW level were significantly lower in the acute appendicitis group (p < 0.001, p < 0.001, p = 0.001, respectively). RDW, leukocyte count, and CRP had a sensitivity and specificity of 47% and 67%; 91% and 74%; and 97% and 41%, respectively in acute appendicitis. RDW was not correlated with CRP and leukocyte levels. However, we found a correlation between CRP and leukocyte levels.

**Conclusion:**

RDW level was lower in patients with acute appendicitis. The magnitude of difference in RDW seen between acute appendicitis and controls was so slight as to be of no utility in diagnostic testing.

## Introduction

Acute appendicitis (AA) is the most common surgical abdominal emergency [1]. Rapid diagnosis is important, because increased time between onset of symptoms and surgical intervention is associated with increased risk of appendiceal perforation and therefore potential peritonitis, sepsis, and death [2]. However, the rate of negative appendectomy (when appendectomy is performed, but the appendix is found to be normal on histological evaluation) ranges from 5% to 42%, and this can be associated with considerable morbidity [1-4]. Clinical diagnosis can be challenging, particularly in the early stages of appendicitis when clinical manifestations may be quite non-specific or atypical. Different elements of history, examination, and laboratory findings have varying predictive power in the diagnosis of appendicitis, and algorithms and scoring systems for clinical evaluation exist, but appendicitis can nevertheless be easily missed [1,3].

The preoperative laboratory tests can be performed easily in primary healthcare settings and often aid primary clinicians with decision making about patients with clinically suspected AA. Several parameters for the diagnosis of AA have been investigated in the literature [5]. RDW, a measure of heterogeneity in the size of circulating red blood cells, is a component of the standard complete blood count and calculated as a percentage of the standard deviation of the red cell volume divided by the mean corpuscular volume. It has been reported that RDW level has clinical implications in various pathologies such as inflammatory bowel disease, celiac disease, pulmonary embolism, and coronary artery disease [6-10]. In addition, its predictive role has been shown in inflammatory and infectious pathological diseases including acute pancreatitis, bacteremia, sepsis, and septic shock [11-13]. In the present study we aimed to seek whether RDW level is important in the diagnosis of AA. No studies in literature have examined this subject before. In addition, it was aimed to show the relationship of RDW level with leukocyte count and CRP level.

## Materials and method

The main analysis in this study was the comparison of the difference RDW measurements between acute appendicitis and control groups. In healthy individuals RDW levels have been reported as 11.6% and 15.5% with a standard deviation of approximately 1.3%. A 0.6% difference in the mean RDW values was determined to represent a significant difference between acute appendicitis and control groups. Given these assumptions and assuming that two-sample independent t test was to be used for comparison of means, it was determined that both groups had to include at least 123 test subjects to achieve a power level of 90%. Sample sizes were calculated by using the Minitab statistical package software (Release 14). This study was conducted via retrospective assessment of hospital records of the adult patients who were operated for acute appendicitis in Baskent University, Konya Research and Application Center, between January 2010 and February 2013 and had a pathology report that confirmed the diagnosis of acute appendicitis. A total of 590 patients were included in the AA group. The patients in the control group were selected from healthy adults of similar age who applied to check-up clinic and had no active complaint, chronic disease, or abnormal physical examination. Age, gender, leukocyte count, and CRP and RDW levels were recorded. This study is a case controlled retrospective clinical study.

### Laboratory measurements

WBC counts were determined using an electronic cell counter (Cell-Dyne 3700, Abbott, Abbott Park, IL, USA). Serum CRP levels were measured by spectrophotometric methods (Abbott Aeroset, Tokyo, Japan). The expected RDW values in our laboratory ranged between 11.6% and 15.5%.

### Statistical analysis

Statistical analyses were performed with SPSS software. The groups were compared using the *t* test for continuous variables and chi-square test for categorical variables. Mann–Whitney *U* test was used to compare nonhomogeneous groups in pairs. A simple correlation test (Spearman’s test) was used to observe the correlation between the RDW and other variables. Numeric values were expressed as means ± SD. A *P* value less than .05 was considered statistically significant.

## Results

**Figure 1:**
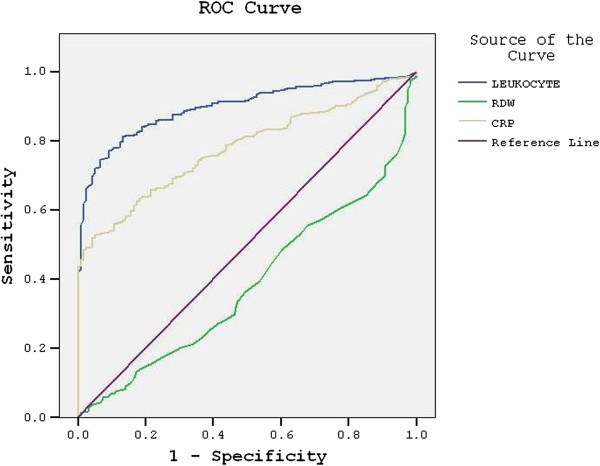
Receiver operating characteristic (ROC) curve of red cell distribution width (RDW), leukocyte, and C-reactive protein (CRP).

A total of 590 patients were included in the AA group and 121 patients were included in the control group, making up a total of 711 subjects. No significant difference was observed between the AA and control groups with respect to age and gender p > 0.05 (Table 1). The mean leukocyte count was 13.5 ± 4.5 (×10^3^/mm^3^) in the AA group and 7.5 ± 2 (×10^3^/mm^3^) in the control group. The leukocyte count was significantly higher in the AA group (p < 0.001). The mean CRP level was 48.8 ± 73.6 mg/dL in the AA group and 4.6 ± 4.7 mg/dL in the control group. CRP level in the AA group was significantly higher compared with the control group (p < 0.001). The mean RDW level was 15.4 ± 1.5% in the AA group and 15.9 ± 1.4% in the control group. RDW level was significantly lower in the AA group compared with the control group (p = 0.001) (Table 1). Receiver operating characteristic curve analysis suggested that the best cutoff point for RDW in the diagnosis of AA was 15.6%, which had a sensitivity of 47% and a specificity of 67%, (area under curve [AUC]: 0,62; Figure 1). Receiver operating characteristic curve analysis suggested that the best cutoff point for leukocyte count in the diagnosis of AA was 10.4 (×10^3^/mm^3^), which had a sensitivity of 91% and a specificity of 74% (area under curve [AUC]: 0.9; Figure 1). Receiver operating characteristic curve analysis suggested the best cutoff point for CRP level in the diagnosis of AA was 27.1 mg/dL, which had a sensitivity of 97% and a specificity of 41% (area under curve [AUC]: 0.77; Figure 1). RDW was not correlated with CRP and leukocyte levels. However, we found a correlation between CRP and leukocyte levels (Table 2).

**Table 1:** Comparison of the demographic features and leukocyte count, CRP, and RDW levels of the subjects in the acute appendicitis and the control groups

	**Acute appendicitis (n = 590)**	**Control group (n = 121)**	***p***
**Male/female**	332/258	69/52	.82
Age (y)*	36.7 ± 12.2	35.2 ± 8.1	.67
**Leukocyte (× 10**^**3**^/mm3)*	13.5 ± 4.5	7.5 ± 2	<0.01
CRP (mg/L)*	48.8 ± 73.6	4.6 ± 4.7	<0.01
RDW (%)*	15.4 ± 1.5	15.9 ± 1.4	0.01

*Values are means±standard deviation.

*Abbreviations*: *CRP* C-reactive protein, *RDW* red cell distribution width.

**Table 2:** Correlation analysis of leukocyte, CRP, and RDW levels in patients with acute appendicitis

**Parameters**	**Correlation coefficient (r)**	***P value***
**Leukocyte - RDW**	-0.031	.44
**Leukocyte - CRP**	0.21	<0.01
**CRP – RDW**	-0.065	.11

*Abbreviations*: *CRP* C-reactive protein, *RDW* red cell distribution width.

## Discussion

A parameter with ability to establish the diagnosis of acute appendicitis has always been a center of attention for physicians. Many different parameters have been examined or are under active investigation for that purpose. The pathophysiology of acute appendicitis is characterized by the mucosal ischemia of the appendix that results from ongoing mucus secretion from the appendiceal mucosa distal to an obstruction of the lumen, elevating intraluminal and, in turn, venous pressures. Once luminal pressure exceeds 85 mmHg, venules that drain the appendix become thrombosed and, in the setting of continued arteriolar in flow, vascular congestion and engorgement of the appendix become manifest [5]. Infection is added to the inflammation of appendicitis.

WBC count is most frequently used to diagnose AA. Several reports have suggested that an elevated WBC count is usually the earliest laboratory measure to indicate inflammation of the appendix, and most patients with AA present with leukocytosis [14,15]. We found that WBC count was significantly higher in AA. In various studies, the range of sensitivity and specificity of WBC in the diagnosis of AA have been reported 67%- 97.8% and 31.9%-80%, respectively [16]. Similar to the literature, the present study found that the sensitivity and specificity of leukocyte level were 91% and 74%, respectively.

CRP is a sensitive acute phase protein that lacks specificity due to increased levels in all acute inflammatory processes. Its concentration increases with the duration and extent of the inflammation. In a meta-analysis examining the accuracy of CRP level in the diagnosis of AA, Hallan and Asberg found a wide range of sensitivity (40%-99%) and specificity (27%-90%) [17]. Similar to the literature, this study found a sensitivity of 97% and a specificity of 41% for CRP in the diagnosis of acute appendicitis. Among the assessed parameters, CRP had the highest sensitivity and the lowest specificity.

RDW is commonly used to discriminate between microcytic anemia’s due to iron deficiency and those due to thalassemia or hemoglobinopathies [7]. Increased RDW levels are related to impaired erythropoiesis or erythrocyte degradation [7]. The typical reference range spans between 11.6 and 15.5%. Recent studies have demonstrated that higher RDW levels, even within the normal reference range, were associated with unfavorable clinical outcomes in patients with heart failure, coronary artery disease, pulmonary hypertension, diabetes mellitus, and stroke independent of hemoglobin values [8-10,18,19]. Furthermore RDW has been studied as a surrogate marker in many pathological conditions such as rheumatoid arthritis, inflammatory bowel disease, colon cancer, and celiac disease [6,20,21]. Although the exact pathophysiological basis of this relationship is unclear, chronic inflammation, aging, malnutrition, and anemia are proposed underlying factors in this topic [10,22].

In a patient with acute pancreatitis, RDW level at the presentation has been reported to be an independent risk factor for mortality [12]. Similarly, RDW level has also been found to be a predictor for mortality in bacteremia and septic shock [11,13]. An increased RDW level has been reported in these inflammatory and infectious pathologies. Elevated RDW can result from any disease process that causes the premature release of reticulocytes into the circulation. Elevations in RDW have been shown to be associated with elevated inflammatory markers, such as CRP, erythrocyte sedimentation rate, and interleukin 6 [13,23,24]. Proinflammatory cytokines of sepsis (tumor necrosis factor A, interleukin 6, and interleukin 1b) have been shown to directly and negatively affect the survival of red blood cells in the circulation, promote deformability of the red blood cell membrane, and suppress erythrocyte maturation. These inflammatory mediators of sepsis can thus lead to newer, larger reticulocytes to enter the peripheral circulation, and thus increase RDW [13]. Unlike the above-mentioned studies, we found a significantly lower, albeit within normal limits, RDW level in patients with acute appendicitis compared with subjects in the control group. This finding may be the result of greater RDW level in chronic inflammatory diseases compared to that in acute conditions. A strong correlation of RDW with inflammatory markers, CRP and sedimentation rate has also been observed [13,23,24]. In our study, on the other hand, RDW was not correlated with CRP and leukocyte count.

In conclusion, RDW level was lower in patients with acute appendicitis. No studies in literature have examined RDW level in acute appendicitis before. The magnitude of difference in RDW seen between AA and controls was so slight as to be of no utility in diagnostic testing. We think that further prospective, multicenter studies with a large sample size are needed in this field.

## Competing interests

The authors declare that they have no competing interests and no funding statement.

## Authors’ contributions

Study concept and design: HN, ET and EK. Analysis and interpretation of data: HN, ET, EK, TT and KK. Drafting of the manuscript: HN, ET and EK. All authors read and approved the final manuscript.
